# Photophysics of 2D Organic–Inorganic Hybrid Lead Halide Perovskites: Progress, Debates, and Challenges

**DOI:** 10.1002/advs.202001843

**Published:** 2021-01-29

**Authors:** Zhixing Gan, Yingchun Cheng, Weijian Chen, Kian Ping Loh, Baohua Jia, Xiaoming Wen

**Affiliations:** ^1^ Center for Future Optoelectronic Functional Materials School of Computer and Electronic Information/School of Artificial Intelligence Nanjing Normal University Nanjing 210023 China; ^2^ College of Materials Science and Engineering Qingdao University of Science and Technology Qingdao 266042 China; ^3^ Key Laboratory of Flexible Electronics (KLOFE) & Institute of Advanced Materials (IAM) Nanjing Tech University 30 South Puzhu Road Nanjing 211816 China; ^4^ Centre for Translational Atomaterials Faculty of Science Engineering and Technology Swinburne University of Technology John Street Hawthorn VIC 3122 Australia; ^5^ Australian Centre for Advanced Photovoltaics School of Photovoltaic and Renewable Energy Engineering UNSW Sydney Kensington NSW 2052 Australia; ^6^ Department of Chemistry and Centre for Advanced 2D Materials and Graphene Research Centre National University of Singapore Singapore 117543 Singapore

**Keywords:** multiquantum‐well, perovskites, photoluminescence, photophysics

## Abstract

2D organic–inorganic hybrid Ruddlesden–Popper perovskites (RPPs) have recently attracted increasing attention due to their excellent environmental stability, high degree of electronic tunability, and natural multiquantum‐well structures. Although there is a rapid development of photoelectronic applications in solar cells, photodetectors, light emitting diodes (LEDs), and lasers based on 2D RPPs, the state‐of‐the‐art performance is far inferior to that of the existing devices because of the limited understanding on fundamental physics, especially special photophysics in carrier dynamics, excitonic fine structures, excitonic quasiparticles, and spin‐related effect. Thus, there is still plenty of room to improve the performances of photoelectronic devices based on 2D RPPs by enhancing knowledge on fundamental photophysics. This review highlights the special photophysics of 2D RPPs that is fundamentally different from the conventional 3D congeners. It also provides the most recent progress, debates, challenges, prospects, and in‐depth understanding of photophysics in 2D perovskites, which is significant for not only boosting performance of solar cells, LEDs, photodetectors, but also future development of applications in lasers, spintronics, quantum information, and integrated photonic chips.

## Graphical abstract

This review highlights the special photophysics of 2D perovskites that is fundamentally different from the conventional 3D congeners, which is significant for not only boosting performance of solar cells, light emitting diodes, photodetectors, but also future development of applications in lasers, spintronics, quantum information, and integrated photonic chips.

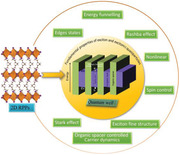

## 1 Introduction

Given the background that hybrid lead halide perovskites (LHPs) have become one of the most popular semiconductors in optoelectronics area over the past years,^[^ ^1^, ^2^, ^3^, ^4^, ^5^ ^]^ there have been explosive investigations on 2D organic–inorganic hybrid Ruddlesden–Popper perovskites (RPPs). 2D layered RPPs were discovered before the rise of ordinary organic–inorganic LHP of ABX_3_. In the 1990s, Mitzi et al. originally introduced layered organic–inorganic hybrid perovskites (C_4_H_9_NH_3_)_2_(CH_3_NH_2_)*_n_*_−1_Sn*_n_*I_3_ *_n_*_+1_, which are analogous to the Ruddlesden–Popper phase of K_2_NiF_4_ material.^[^ ^6^ ^]^ For the 2D RPPs, inorganic perovskite layers are intercalated with bulky organic cations acting as the spacers. The general chemical formula of such 2D RPPs is (RNH_3_)_2_A*_n_*_−1_M*_n_*X_3_ *_n_*_+1_ (*n* = 1, 2, 3, 4…), where RNH_3_ is a large bulky aliphatic or aromatic alkylammonium spacer cation, typically, *n*‐butylammonium (*n*‐BA) and 2‐phenylethylammonium (PEA), A is monovalent organic cation, such as CH_3_NH_3_^+^ (MA^+^) and HC(NH_2_)_2_^+^ (FA^+^), M is a divalent metal cation, such as Pb and Sn, X is a halide anion (X = Cl, Br, I), and *n* represents the number of [MX_6_]^4−^ octahedral layers within each period.^[^ ^7^ ^]^

Compared to ordinary 3D organic–inorganic LHPs with inherent instabilities, the 2D layered RPPs have recently attracted significant attention owing to their improved environmental stability when subjected to light, humidity, and heat stress.^[^ ^8^, ^9^ ^]^ Ion migration in a 3D perovskite is the source of many instabilities, such as photocurrent hysteresis and giant switchable photovoltaic effect, which can accelerate the degradation of perovskite‐based electronic devices.^[^ ^10^, ^11^, ^12^, ^13^, ^14^, ^15^ ^]^ The intrinsically better stability of 2D RPPs is largely attributed to the absence of ion migration.^[^ ^16^ ^]^ Compared to 3D perovskites, the formation of an ion vacancy in 2D RPPs requires more energy, suppressing ion migration. Hydration of 3D perovskite surface induced by moisture can cause the poor contact, resulting in performance loss of optoelectronic devices.^[^ ^14^, ^15^ ^]^ Since the organic spacers can suppress the penetration of moisture to the perovskites, the 2D layered LHPs are also expected to exhibit improved moisture stability.^[^ ^17^, ^18^, ^19^ ^]^ Thus, extremely smooth and clean surface can be acquired in 2D layered LHPs via mechanical exfoliation, which will significantly enhance the optoelectronic performance. It is reported that the smooth 2D perovskite less sensitive to ambient moisture exhibits a quite low dark current, outperforming the rough surface by 23.6 times in terms of photodetectivity.^[^ ^19^ ^]^ Consequently, the unencapsulated solar cells based on 2D RPPs retain over 60% of their maximum efficiency for over 2250 h under constant, standard illumination (AM1.5G), and the devices exhibit a greater tolerance to relative humidity of 65% than their 3D counterparts.^[^ ^8^ ^]^ Very recently, Ren et al. developed a new 2D RPPs by using 2‐(methylthio)ethylamine hydrochloride (MTEACl) as the bulky alkylammoniums spacer.^[^ ^9^ ^]^ The (MTEA)_2_(MA)_4_Pb_5_I_16_ (*n* = 5) RPPs showed enhanced charge transport and improved stabilization due to weakened van der Waals interactions. Significantly improved efficiency and stability were achieved in the solar cells based on this new type of 2D RPPs. A power conversion efficiency as high as 18.06% (17.8% certified) was acquired, where the solar cells showed excellent moisture tolerance for 1512 h (70% relative humidity), thermal stability for 375 h (85 °C), and photostability up to 1000 h (operation at the maximum power point).^[^ ^9^ ^]^

In addition to the excellent stability, the electronic structures of 2D RPPs are more flexible, compared to their 3D counterparts. The band gaps of 2D RPPs can be tuned by the number of inorganic layers (*n* value) and the inserted long‐chain ligands, in addition to the halogen component. For example, the bandgap of PEA_2_(MA)*_n_*_−1_Pb*_n_*I_3_ *_n_*_+1_ changes from ≈2.6 eV for *n* = 4, to 2.7, 2.9, 3.1 eV for *n* = 3, 2, 1, respectively.^[^ ^20^ ^]^ Besides their superior stability and high degree of tunability, another intriguing property is the natural multiquantum‐well (MQW) structures of the 2D RPPs.^[^ ^21^, ^22^, ^23^, ^24^, ^25^, ^26^, ^27^, ^28^ ^]^ The barrier of organic ligands and the quantum well (QW) of inorganic octahedral layers have very different dielectric constants.^[^ ^24^ ^]^ The Coulomb interaction in the QW is strictly screened by the presence of the organic barrier because of the well‐known dielectric confinement effect caused by the high contrast in dielectric constants.^[^ ^24^, ^25^, ^26^ ^]^ Therefore, the electron–hole interaction within the exciton is very strong, resulting in huge oscillator strengths, large exciton binding energies, and novel 2D quantum confinement effect. Moreover, the electronic, optical, and magnetic properties also can be tailored accordingly.^[^ ^27^ ^]^

On the basis of their excellent stability, flexible bandgap, and MQW effect, the 2D RPPs have exhibited abundant optoelectronic properties and promising applications in solar cells,^[^ ^27^ ^]^ photodetectors,^[^ ^28^ ^]^ light‐emitting devices (LEDs),^[^ ^29^ ^]^ lasers,^[^ ^30^ ^]^ laser cooling,^[^ ^31^ ^]^ and strong optical nonlinearities.^[^ ^8^ ^]^ The 2D RPPs generally exhibit high quantum efficiency (QE), for examples, respectable power conversion efficiency of 12.51% for solar cell^[^ ^32^ ^]^ and high external quantum efficiency (EQE) for LEDs.^[^ ^33^ ^]^ In particular for the LEDs, a maximum luminance of 2480 cd m^−2^ at 490 nm is achieved based on the 2D RPPs with record‐high photoluminescence (PL) quantum yield (QY) of 88%.^[^ ^34^ ^]^ By using quasi‐2D RPPs as the active layer, a LED with an EQE of 8.8% and radiance of 80 W sr^−1^ m^−2^ at the near‐infrared wavelengths has also been demonstrated.^[^ ^29^ ^]^ The EQE was rapidly improved to 11.7%. And a higher radiance of around 82 W sr^−1^ m^−2^ is achieved at 3.6 V.^[^ ^35^ ^]^ By applying triplet management strategies, Qin et al. achieved stable green lasers from quasi‐2D perovskite under continuous wave (CW) optical pumping in air at room temperature using a distributed‐feedback cavity with a high‐quality factor.^[^ ^36^ ^]^

Although there is a rapid development of photoelectronic applications based on 2D RPPs, the state‐of‐the‐art performance is far from mature due to the limited understanding on the fundamental physics, especially special photophysics in carrier dynamics, excitonic fine structures, excitonic quasiparticles, and spin‐related effect. Thus, there is a huge potential to improve the performance of solar cells and LEDs based on the 2D RPPs by enhancing the understanding on fundamental photophysics. Hitherto, there are only few review articles spotting light on photophysics of 2D perovskites. Pedesseau et al. discussed the excitonic properties and possible Rashba effect related to the loss of inversion symmetry.^[^ ^37^ ^]^ Recently, 2D RPPs for optoelectronics have been reviewed by Chen et al.^[^ ^7^ ^]^ Summary on the structures, growths, and optoelectronic performances of 2D RPPs with different chemical components are outlined in their review.^[^ ^7^ ^]^ Up‐to‐date progress on optical and optoelectronic properties of 2D RPPs was reviewed by Gao et al.^[^ ^38^ ^]^ and Wang et al.^[^ ^39^ ^]^ Recently, a comprehensive review covering synthesis methods, optical properties, and device demonstrations with a focus on thickness and dimensionality controlled properties was presented by Leng et al.^[^ ^40^ ^]^ However current reviews mainly summarize common optical properties and their corresponding applications mainly limited to LEDs and PV in a general manner without a focus on the fundamental photophysics.

Since there are growing reports on the newly emerging novel photophysics, such as quasiparticle dynamics, transient photophysics, and spin‐related properties, the topic on 2D RPPs has drawn increasing research effort driven by the need to understand the fundamental physical process of light interaction with the materials, which is critical for significant improvement of device performance and will deserve much more attention in the future. This encourages us to review the recent progress on the novel photophysics of 2D RPPs in this work.

**Figure 1:**
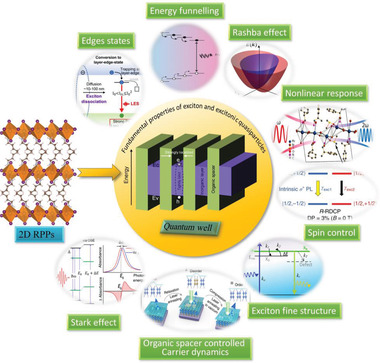
Schematic illustration of the key photophysical processes in 2D organic–inorganic hybrid lead halide perovskites. ("Edge states": Reproduced with permission.^[^ ^89^ ^]^ Copyright 2017, American Association for the Advancement of Science. "Energy funnelling": Reproduced with permission.^[^ ^29^ ^]^ Copyright 2016, Springer Nature. "Rashba effect": Reproduced with permission.^[^ ^117^ ^]^ Copyright 2017, American Association for the Advancement of Science. "Nonlinear response": Reproduced with permission.^[^ ^132^ ^]^ Copyright 2018, American Chemical Society. "Stark effect": Reproduced with permission.^[^ ^153^ ^]^ Copyright 2016, American Association for the Advancement of Science. "Exciton fine structure": Reproduced with permission.^[^ ^136^ ^]^ Copyright 2019, Wiley‐VCH GmbH. "Organic spacer controlled Carrier dynamics": Reproduced with permission.^[^ ^148^ ^]^ Copyright 2018, Springer Nature.)

As schemed in **Figure** **1**, this review starts from the fundamental physics of exciton and excitonic quasiparticle behaviors of 2D RPPs (Section 2). Novel photophysics that arising from these special 2D structures are then reviewed in Section 3. The internal exciton dissociation through edge states and energy funneling between different nanodomains that uniquely observed in 2D perovskites are discussed in Sections 3.1–3.2. In the following Sections 3.3–3.6, the structural symmetry related effects, including progresses on Rashba splitting, nonlinear optical properties, and spin control are reviewed. Section 3.7 focus on the organic cation related hot carrier cooling and bandgap‐modulation effects. Section 3.8 reviews the progress on anomalous Stark effect. To facilitate a better flow of the review, the terminology is defined as follow: Generally, the RPPs with *n* > 1 is named as quasi‐2D RPPs. Here, for simplicity of description, all RPPs with different *n* values are called 2D without a distinction. The RPPs, especially solution‐processed films, usually contain grains with different layer number *n*, the average *n* is denoted as 〈*n*〉.

## 2 Fundamental Properties of Carrier, Exciton, and Excitonic Quasiparticles

### 2.1 Strong Excitonic Binding Energy

Due to the huge energy difference and high contrast in dielectric constants between the bandgap of the inorganic layer (QW) and the organic layer (barrier), the Coulomb interaction between photogenerated electron–hole is very strong, resulting in significant oscillator strengths and very large exciton binding energies (*E*_b_, typically few hundreds of meV).^[^ ^24^ ^]^ The quantum confinement leads to strongly bound excitons with binding energy up to 300–400 meV for phenylethylammonium (PEA = C_6_H_4_CH_2_CH_4_NH_3_) lead iodide (PEA_2_PbI_4_) RPPs,^[^ ^28^ ^]^ which is evidently larger than that of 3D LHP. As a comparison, the reported exciton binding energy for iodine‐based 3D perovskites is in the range from 2 to 50 meV,^[^ ^41^ ^]^ and 67–150 meV for bromine‐based 3D perovskites.^[^ ^42^ ^]^ Furthermore, the divergence between excitonic binding energy of 2D and 3D is filled by quasi‐2D perovskites with *n* > 1.^[^ ^43^, ^44^, ^45^ ^]^ Excitons in 2D RPPs are anisotropic. For in‐plane directions along the inorganic perovskite layer, the excitons remain delocalized, exhibiting Wannier‐like behavior.^[^ ^46^, ^47^ ^]^ However, along the direction perpendicular to the inorganic perovskite layer, excitons are localized as Frenkel‐like behavior, with high oscillator strengths that are suitable for the generation of high exciton densities.

The most direct consequence of strong excitonic binding is efficient radiative recombination. A variety of applications in luminescence are benefited from the large exciton binding energy and oscillator strength. Luminescence streaming from excitonic recombination in the 2D MQWs exhibits a much higher decay rate and efficiency than that from bimolecular recombination in their 3D counterparts. Its first‐order exciton recombination can effectively compete with defect trapping over a broad range of injected charge carrier densities. Therefore, a near invariant high PL QY of around 60% can be acquired when carrier densities at the thin wells are lower than 10^16^ cm^−3^.^[^ ^34^ ^]^

**Figure 2:**
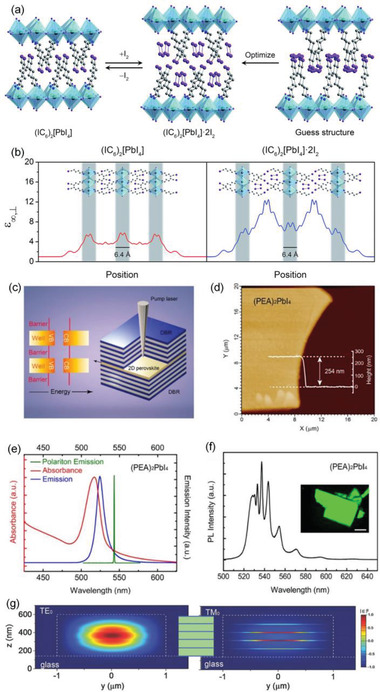
a) Scheme illustrating the intercalation of I_2_ in single‐crystal of 2D (IC_6_)_2_[PbI_4_]. Dark green, purple, blue, and grey spheres represent Pb, I, N, and C atoms, respectively. b) Calculated high‐frequency dielectric profiles perpendicular to the direction of layer propagation before (left) and after (right) intercalation of I_2_. Reproduced with permission.^[^ ^48^ ^]^ Copyright 2017, The Royal Society of Chemistry. c–f) Illustration of polariton emission. c) Schematic of energy‐band structure of 2D RPPs (left) and a 2D microcavity, which is consisted of a 2D RPPs crystal inserted in two planar DBRs (right). d) Atomic force microscopy image of an exfoliated 2D (PEA)_2_PbI_4_ RPP crystal after microcavity fabrication. e) Room temperature absorption and PL spectra of a thin 2D (PEA)_2_PbI_4_ RPP flake, and polariton emission of a 2D RPP embedded into the microcavity. f) Room temperature PL spectrum of a thick 2D RPP crystal on a silicon substrate. Inset: the corresponding fluorescence microscopy image, scale bar = 10 µm. Reproduced with permission.^[^ ^70^ ^]^ Copyright 2018, American Chemical Society. g) Illustration of photon confinement. In the middle, the green and blue layers represent the perovskite layers with refractive index of 2.3 and 1.6, respectively. The left and right panels represent TE_0_ and TM_0_ optical field distributions in the multilayered RP perovskite from finite‐difference time‐domain (FDTD)‐simulation. Reproduced with permission.^[^ ^76^ ^]^ Copyright 2018, Wiley‐VCH GmbH.

However, on the other hand, for ideal solar‐cell absorbers, weak binding energy between electrons and holes is favorable for charge carriers to easily separate and transport toward their respective charge collectors. Therefore, control over the exciton binding energy in 2D RPPs is required to enable their applications in a broad range of optoelectronic technologies. Smith et al. demonstrated that 2D RPPs can be stabilized by inserting small molecules into the binding spacers,^[^ ^48^ ^]^ and show that I_2_ intercalation could lead to a more polarizable organic layers compared to the inorganic layers, which significantly reduces the dielectric confinement of excitons generated in the inorganic layers (**Figure** **2**a). As shown in Figure 2b, the *ε*_∞,⊥_ dielectric constant associated with the organic layers dramatically increase from 3.7 to 11.1 after I_2_ intercalation.^[^ ^48^ ^]^ Therefore, I_2_ intercalation significantly decreases the dielectric confinement of excitons in the inorganic layers by enhancing screen of electric field lines in the organic layer. The *E*_b_ values for (C_6_H_13_NH_3_)_2_[PbI_4_] and (C_6_H_13_NH_3_)_2_[PbI_4_]⋅2I_2_ resulting from calculations are 288 and 171 meV, respectively, which is consistent with the experimental results that *E*_b_ value of (C_6_H_13_NH_3_)_2_[PbI_4_] decreases from 230 to 180 meV after I_2_ intercalation.^[^ ^48^ ^]^ It should be noted that, for most 2D RPPs, the length of organic cation of the 2D RPPs is notably smaller than the barrier thickness of conventional artificial MQWs.^[^ ^49^ ^]^ In these 2D RPPs, the exciton Bohr radius has been estimated to be 1.7 nm in the plane of the layers, equal to 3 or 4 unit cells, thus the envelope functions of the neighbor inorganic layers are not strictly separated. Therefore, 2D RPPs should be regarded as MQWs with caution.

### 2.2 Carrier/Exciton Mobility and Transport

High mobility and long transport distance are crucial for better photoexcited carrier extraction efficiency.^[^ ^50^ ^]^ In 3D LHPs, very long diffusion lengths, even exceeding microns have been reported.^[^ ^51^, ^52^, ^53^, ^54^ ^]^ However, in 2D RPPs, the quantum confinement and dielectric confinement hinder the carrier mobility. The mobility of 2D RPPs substantially decreases as n value decreases due to the quantum confinement. For (BA)_2_(MA)*_n_*_−1_Pb*_n_*I_3_ *_n_*_+1_, the mobility values are 2 × 10^−3^, 8.3 × 10^−2^, and 1.25 cm^2^ V^−1^ s^−1^ at 77 K when n = 1, 2, and 3, respectively.^[^ ^55^ ^]^ Thereby, Milot et al. indicated that the increase of quantum confinement effects and exciton binding energy would lead to the decrease of charge carrier mobility and increase of bimolecular and Auger recombination rates.^[^ ^56^ ^]^ By balancing the trade‐off between trap reduction, quantum confinement, and layer orientation, highly effective carrier mobility of nearly 10 cm^2^ V^−1^ s^−1^ and long carrier diffusion lengths of 2.5 µm were reached. To improve mobility, Cheng et al. largely reduced the exciton binding energy by using high dielectric‐constant organic spacers.^[^ ^57^ ^]^ Time‐of‐flight measurements demonstrated that the electron and hole mobilities are ≈11.1 and 9.5 cm^2^ V^−1^ s^−1^, respectively. In addition to reducing quantum confinement, Zhao et al. demonstrated that the long‐distance carrier transport in 2D RPPs could be enabled by a trap‐mediated charge transport process.^[^ ^58^ ^]^ A long‐lived (hundreds of nanoseconds) and nonluminescent electron−hole separated state is produced by trap‐induced exciton dissociation. The carrier diffusion distances for *n* = 2–4 2D RPPs are as long as 2−5 µm.

The temperature‐dependent mobility also has been reported. Ziegler et al. directly monitored the diffusion of excitons in hBN‐encapsulated 2D RPPs through ultrafast emission microscopy from liquid helium (5 K) to room temperature (290 K).^[^ ^59^ ^]^ At room temperature, the diffusivity of exciton is 1 cm^2^ s^−1^, equivalent to a high exciton mobility of 40 cm^2^ V^−1^ s^−1^. At 50 K, the diffusivity and effective mobility of the free exciton transport are as high as 3 cm^2^ s^−1^ and 1000 cm^2^ V^−1^ s^−1^, respectively. When approaching liquid helium temperature in experiment, a nonlinear regime of anomalous diffusion showed a rapidly expanding exciton cloud and diffusivities up to 30 cm^2^ s^−1^.

### 2.3 Strong Exciton–Phonon Interactions

Another consequence from the large exciton binding energy and oscillator strength is the strong exciton–phonon interactions. The exciton–phonon interaction of 2D PEA lead iodide RPPs^[^ ^60^ ^]^ is found to be more than one order of magnitude higher than that in GaAs QWs^[^ ^61^ ^]^ based on the temperature‐dependent PL measurements. The longitudinal optical (LO) phonon energy (*E*_LO_) was determined to be 14 meV and the exciton coupling with the optical phonon strength (Γ_LO_) was estimated to be on the order of 70 meV. The acoustic phonon scattering parameter (Γ_ac_) is equal to 0.03 ± 0.01 meV K^−1^. As a result, PL emission lines have to be interpreted in the framework of a polaron model. Similar conclusion was also achieved in other 2D LHP, such as BA lead iodide (BA)_2_PbI_4_ (BA = CH_3_(CH_2_)_3_NH_3_) and hexylammonium (HA) lead iodide (HA)_2_PbI_4_ (HA = CH_3_(CH_2_)_5_NH_3_).^[^ ^60^ ^]^ At low temperatures (<100 K), the PL linewidth broadening is due to acoustic phonon scattering at 0.026 meV K^−1^, whereas at high temperatures, LO phonon–exciton coupling is the dominant mechanism that broadens the PL. The phonon–exciton coupling parameters of three typical 2D RPPs are summarized in **Table** **1**.

**Table 1:** Typical phonon–exciton coupling parameters.^[^ ^60^ ^]^

	LO phonon–exciton coupling		Acoustic phonons scattering Γ_ac_ [meV K^−1^]
	*E*_LO_ [meV]	Γ_LO_ [meV]	
(PEA)_2_PbI_4_	14	70	0.03 ± 0.01
(BA)_2_PbI_4_	10.9	34.2 ± 0.3	0.026 ± 0.003
(HA)_2_PbI_4_	17	55.8 ± 0.4	0.026 ± 0.003

PL spectroscopy is a powerful tool to study the exciton–phonon interactions. For example, electron–phonon couplings of 2D RPPs HC(NH_2_)_2_PbI_3_ and HC(NH_2_)_2_PbBr_3_ are investigated by the temperature dependent PL line broadening.^[^ ^62^ ^]^ It was found that scattering from LO phonons via the Fröhlich interaction is the dominant source of electron–phonon coupling near the room temperature, with negligible scattering via acoustic phonons. The interacting energies of LO phonon modes are determined to be 11.5 and 15.3 meV, and Fröhlich coupling constants are about 40 and 60 meV for the lead iodide and bromide perovskites, respectively. Similarly, high electron–phonon coupling near the room temperature was also discovered to be dominated by LO phonons via the Fröhlich interaction in the (PA)_2_(MA)_2_Pb_3_Br_10_ (propylammonium, PA = CH_3_(CH_2_)_2_NH_3_^+^) hybrid perovskite.^[^ ^63^ ^]^ The strong exciton–phonon coupling in 2D RPPs can accelerate polariton relaxation, which is beneficial for realizing polariton lasing with very low threshold.^[^ ^64^, ^65^ ^]^ It could add interesting opportunities for the development of broadband, short‐pulsed lasers.

For applications in LEDs, strong exciton–phonon coupling in 2D QWs generally is not an advantage because it causes linewidth broadening and may result in undesired asymmetric line shape. Fortunately, in the 2D RPPs, the exciton–phonon coupling induced broadening can be suppressed by changing the organic ligands. Very recently, Gong et al. demonstrated that bright blue emission can be realized in 2D RPPs by reducing the electron–phonon interactions^.[^ ^66^ ^]^ Resonance Raman spectra and deformation potential analysis show that strong electron–phonon interactions result in fast nonradiative decay, lowering the PLQY. Optical phonons, essence of atomic displacements, are important indicators of crystal rigidity. The great rigidity and slow molecular motion are good for emitters. By varying the molecular configuration of the ligands, a notable improvement of PL QY up to 79% was obtained, which is higher compared with other reported 2D RPPs.^[^ ^67^, ^68^ ^]^ Moreover, the narrow linewidth of 20 nm can be reached by controlling crystal rigidity and electron–phonon interactions via optimizing the organic ammonium cations.

### 2.4 Strong Exciton–Photon Coupling

On the basis of the large exciton binding energy and oscillation strength, the confined 2D excitons are also intriguing for the study of excitonic quasiparticles, such as strong exciton–photon coupling, also called as exciton–polaritons. Exciton–polaritons are half‐light, half‐matter quasiparticles formed due to the coupling between excitons and microcavity photons. Thereby, a hybrid state behaving as two separated energy branches (upper polariton branch and lower polariton branch) is formed. The minimum energy difference between these two branches is defined as Rabi‐splitting, $2\hbar \Omega =2{\sqrt{g^{2}-\left(\gamma_{\mathrm{cav}}-\gamma_{\mathrm{exc}}\right)}}^{2}$, which is an index of exciton–photon interaction strength, where *g* is the exciton–photon coupling strength, *γ*_cav_ and *γ*_exc_ are the half‐widths of uncoupled exciton and microcavity resonances.^[^ ^69^ ^]^

As shown in Figure 2c–f, Wang et al. demonstrated strong light–matter couplings with large Rabi splitting (242 meV) and energetic splitting‐to‐line width ratios (>34.2) in the exfoliated 2D (PEA)_2_PbI_4_ RPP at room temperature.^[^ ^70^ ^]^ Due to the strong combination between 2D exciton with Fabry–Pérot cavity modes and Bragg modes of the distributed Bragg reflector (DBRs), the hybridized exciton–polariton states can act as an ultrafast and reversible energy oscillation. To date, the strong coupling between the exciton and the optical mode of a Fabry–Pérot microcavity has been demonstrated at room temperature in both UV^[^ ^71^ ^]^ and visible ranges.^[^ ^72^, ^73^ ^]^ In particular, the demonstration of polariton–polariton interactions may lead to polariton scattering, which would be a fundamental breakthrough for the new devices in the context of the low threshold polariton lasers.^[^ ^74^, ^75^ ^]^

### 2.5 Photon Confinement

Li et al. synthesized (OA)_2_(MA)*_n_*_−1_Pb*_n_*Br_3_ *_n_*_+1_ RPPs by using the long ligand octylamine (OA = CH_3_(CH_2_)_7_NH_2_) as spacers between lead bromide octahedrons layers with OA/MA molar ratio of 1:4.^[^ ^76^ ^]^ The PL spectrum of the RPP layers contains one main peak located at about 530 nm along with several minor peaks at higher photon energies originating from RPPs of *n* = 2–6. Similar to other 2D RPPs,^[^ ^29^, ^35^, ^77^ ^]^ the acquired RPPs are composed of mixed dimensionalities with different *n*‐values. Interesting, the refractive indices of 2D RPPs change with *n*‐values. As calculated by static dielectric constant *ε* using density functional theory, the refractive indices for OA ligands, *n* = 2, and *n* = 3 2D RPPs are 1.01, 1.39, and 1.46, respectively. The refractive index of 2D RPPs based on OA was experimentally determined to be ≈1.43^[^ ^78^ ^]^ while the refractive index of 3D MAPbBr_3_ is about 2.3.^[^ ^79^ ^]^ Thus, the refractive index is smaller for the lower‐dimensional perovskite layers. Therefore, in the microplatelets with mixed RPP layers of different *n* values, the lower‐dimensional region can act as cladding layers while the regions of higher‐dimensional RPPs play the role of waveguiding due to the larger refractive index, resulting in photon confinement (Figure 2g). The interactions between emitted photons and gain medium are enhanced via photon confinement, which will benefit the development of resonant cavity for lasing. Moreover, the anisotropic mode of the laser can be modulated by the photon confinement (Figure 2g). Li et al. demonstrated that the TE field is mainly contained in the higher‐dimensional perovskite layers, while the TM field is majorly contained in the lower‐dimensional ones.^[^ ^76^ ^]^ Consequently, TE‐polarized lasing is observed in the (OA)_2_(MA)*_n_*_−1_Pb*_n_*Br_3_ *_n_*_+1_ RPPs.

### 2.6 Long lived Carriers/Excitons and Long PL Lifetime

The long‐lived PL signal usually implies a long carrier lifetime and a long carrier diffusion length, which is crucial for high‐performance PV. Different photophysical effects, including, defect tolerance (Section 2.7), reversible processes of multiple trapping and detrapping,^[^ ^80^ ^]^ excitonic energy reservoir (Section 3.6), may lead to a long‐lived PL signal. These kinds of effects typically prolong the PL lifetimes to tens of nanoseconds. More than these effects, trap‐induced exciton dissociation may further increase the carrier lifetime to microseconds or even longer.^[^ ^81^, ^82^ ^]^ The carrier lifetimes at electron−hole separated state are found to be as long as 1597 ns.^[^ ^81^ ^]^ Luo et al. synthesized Mn^2+^ homogeneously doped 2D RPPs EA_2_PbBr_4_ (EA = ethylammonium) via a reprecipitation method.^[^ ^82^ ^]^ The resulted Mn^2+^ doped EA_2_PbBr_4_ exhibited a high PL QY of 78% owing to the efficient exciton trapping induced by Mn^2+^ doping and small activation energy. As a result, an extremely long PL lifetime of ≈0.75 ms is observed due to the heavy atom effect.

### 2.7 Defect Tolerance

Traditionally, the trap states are detrimental for the device performances due to their impact on the charge carrier lifetime and effective mobility. Thereby, PV‐grade film and wafers based on conventional semiconductors require ultralow concentrations of impurities and crystalline defects at ppb levels.^[^ ^83^, ^84^, ^85^, ^86^, ^87^ ^]^ Intriguingly, highly efficient PV devices can be acquired based on polycrystalline LHPs films containing a considerable density of point defects, the so‐called defect tolerance.^[^ ^83^, ^84^, ^85^, ^86^, ^87^ ^]^ Trap states are caused by structural defects derived from impurities or interruptions in the crystal lattice. The shallow traps may have little influence on the photophysics. Whereas, the deep traps are considered to bring nonradiative recombination centers, deteriorating efficiency and open‐circuit voltage of PV devices. Pandey et al. investigated the effect of different defects on the band structure of atomically thin 2D perovskites.^[^ ^88^ ^]^ They found that the most common defects only induce shallow or no states in the band structure, suggesting the 2D perovskites exhibit high defect tolerance.

## 3 Novel Photophysics of 2D RPPs

### 3.1 Internal Exciton Dissociation through Edge States

**Figure 3:**
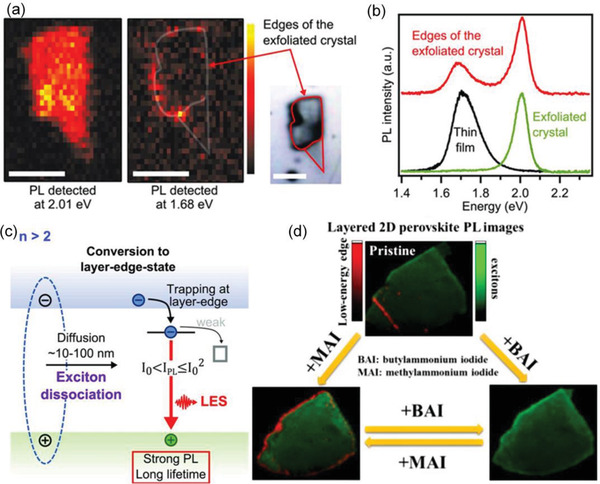
a) PL mappings of a single exfoliated crystal probed at 2.010 eV (left), 1.680 eV (middle), and corresponding microscopic image (right). Scale bar: 10 µm. b) PL spectra of an exfoliated crystal, exfoliated crystal edges, and a thin film. c) Scheme of exciton dissociation through edge states when *n* > 2. Reproduced with permission.^[^ ^89^ ^]^ Copyright 2017, American Association for the Advancement of Science. d) Schematic illustrating the control of the edge states. The crystal edges with exciton dissociation ability can be removed by adding additional BA cations and regenerated by BA‐to‐MA cation exchange. Reproduced with permission.^[^ ^91^ ^]^ Copyright 2019, American Chemical Society.

Exciton dissociation is crucial for charge carriers to be harvested by the current collectors of photoelectronic devices, such as solar cells and photodetectors. Interestingly, there is a special internal exciton dissociation through edge states in 2D RPPs. Blancon et al. discovered that the photophysics of 2D RPPs with *n* > 2 is dominated by lower energy edges states (LES), which dissociates excitons into longer‐lived free‐carriers.^[^ ^89^ ^]^ In their study, the 2D layered (BA)_2_(MA)*_n_*_−1_Pb*_n_*I_3_ *_n_*_+1_ RPP with *n* from 1 to 5 were investigated by confocal spatial PL mapping with ≈1 µm resolution. As shown in **Figure** **3**a,b, majority of the basal plane of the exfoliated 2D RPP exhibits spatially homogeneous band edge emission (2.010 eV), while notable emission at 1.680 eV was also observed from the edges of the exfoliated crystal, which are labeled as LES (Figure 3c). Moreover, time‐resolved PL (TRPL) results suggest that there is a nearly four‐fold increment in the carrier lifetime of the LES as compared to the band edge exciton. Similar results were also observed in the exfoliated crystals with *n* = 4 and 5. However, the LES emission was absent in the cases of *n* = 1 or 2.

These results indicate that, for 2D RPPs with *n* > 2, a part of the photogenerated excitons transport to the edges of the crystal within its diffusion time and convert to LES and then efficiently emits photons at a lower energy level than that of the main exciton. Once the excitons are trapped in these LES, they will be dissociated into longer‐lived free‐carriers with the assistance from the edge state, although the average exciton binding energy is as large as 220 meV. The LES is protected from nonradiative processes, which can significantly boost the efficiency of optoelectronic devices.^[^ ^89^ ^]^ Therefore, these findings pave the way toward the rational design of high‐performance optoelectronic devices based on 2D RPPs. However, the nature of the edge state is still unclear.^[^ ^90^ ^]^ In the 2D materials, edge as termination of their 2D expansion is analogous to the surface of 3D bulk materials, which normally induces detrimental or unwanted effects. The unexpected edge state of 2D RPPs requires further research. Zhao et al. conducted research on the formation mechanism of edge states and their controllability.^[^ ^91^ ^]^ It was found that the edges with exciton dissociation ability are triggered by the loss of BA ligands. Thereby, the edge states can be eliminated by adding additional BA cations and regenerated by BA‐to‐MA cation exchange (Figure 3d).

### 3.2 Energy Funneling

#### 3.2.1 Carriers Concentrated by Energy Funneling

Energy funneling refers to a cascaded energy transfer/carrier diffusion in a funnel‐like gradient bandgap profile.^[^ ^92^ ^]^ Compared to the spontaneous carrier motion, the energy funneling has outstanding features of good directionality and ultralong distance,^[^ ^93^, ^94^ ^]^ which can be utilized to improve the energy‐transfer efficiency. In 3D LHPs, gradient bandgap can be obtained by adjusting the halide compositions. For example, FA lead mixed‐halide (FAPb(Br*_x_*I_1−_ *_x_*)_3_) nanoplatelets (NPs) with gradient bandgap have been fabricated using chemical vapor deposition followed with bromide–iodide substitution by exposure FAPbI_3_ to FABr vapor. In such gradient bandgap structures, photogenerated carriers can effectively transfer and emit in the low bandgap region by energy funnelling.^[^ ^95^ ^]^ Moreover, spatially resolved modulation of the fluorescence of this special NPs can be realized by femtosecond direct laser writing (fs‐DLW).^[^ ^96^ ^]^ Since the NPs possess a gradual bromide–iodide composition along the depth, the replacement of iodide ions by bromide ions can be activated under a controlled laser pulse and fluorescence is thus modulated from red to green.

**Figure 4:**
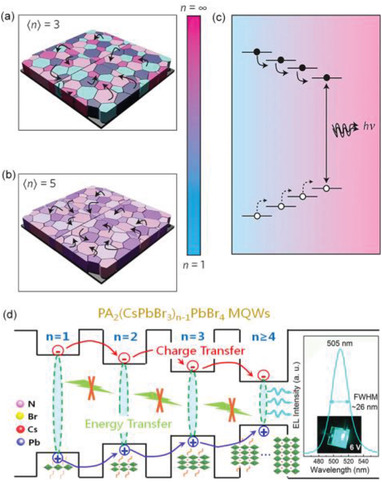
a) Energy funnelling in 2D PEA_2_(CH_3_NH_3_)*_n_*_−1_Pb*_n_*I_3_ *_n_*_+1_ perovskites of 〈*n*〉 = 3. b) Carrier funnelling in 2D multiphase RPPs of 〈*n*〉 = 5. c) Energy diagram of carrier funnelling: carriers converge to narrowest bandgap domains. Reproduced with permission.^[^ ^29^ ^]^ Copyright 2016, Springer Nature. d) Energy funnelling in solution‐processed 2D PA_2_(CsPbBr_3_)*_n_*_−1_PbBr_4_ (propylammonium, PA) is explained by charge‐transfer mechanism, which is responsible for the high performance cyan color RPP LED (inset). Reproduced with permission.^[^ ^106^ ^]^ Copyright 2018, Elsevier Ltd.

Regarding multilayered 2D LHPs, the energy funnelling was pioneeringly proposed by Yuan et al.^[^ ^29^ ^]^ They observed four distinctive peaks in the transient absorption (TA) spectrum and steady‐state absorption spectrum in 2D PEA lead iodide RPPs of 〈*n*〉 = 3. The four peak positions are in good agreement with bandgaps of 2D RPPs with different *n* values. Therefore, the results suggest that the solution‐processed 2D RPP films are not single‐phase, but rather consist of a collection of grains exhibiting a variety of *n* values. In other words, a typical film with an expected *n* value of 〈*n*〉 = 3 also contain *n* = 2, 4, and 5 phases in significant proportions. As well established, the bandgaps of these grains decrease as the *n* values increase. As illustrated in **Figure** **4**a–c, the 2D RPP films are composed of a series of differently quantum‐size‐tuned grains, that, there is a bandgap gradient distribution that funnels carriers to the lowest‐bandgap light‐emitters. Based on TA observations, they concluded that the downward funneling of energy is substantially completed in 100 ps.^[^ ^29^ ^]^

Importantly, the unique energy funneling leads to the concentration of carriers. In this scenario, only the subset of trap states appearing in the lowest‐energy inclusions need to be filled and there is an increased local excitation intensity within small bandgap grains, enabling radiative recombination successfully outcompetes defect trapping and nonradiative recombination, which is absent in 3D perovskites. Hence it is beneficial for high‐efficiency LEDs. Consequently, 2D RPP films with 〈*n*〉 = 3 and 〈*n*〉 = 5 show substantially intensive PL with respective PL QY values of 10.1% and 10.6% under the a low excitation of 6 mW cm^−2^.^[^ ^29^ ^]^ 2D RPP LEDs with a high EQE of 8.8% and a bright radiance of 80 W sr^−1^ m^−2^ were built based on this unique energy funneling process.^[^ ^29^ ^]^

Almost at the same period, energy funneling was also reported in multilayered 1‐naphthylmethylamine (NMA = C_10_H_7_CH_2_NH_2_) lead iodide ((NMA)_2_PbI_4_) RPPs.^[^ ^35^ ^]^ Photo‐induced changes in TA spectra (Δ*A*) of the 〈*n*〉 = 2 RPPs show photobleaching bands at 2.18, 1.95, and 1.64 eV, corresponding to excitonic absorption of domains with *n* = 2, 3, and 4 respectively. The evolution of TA bands shows that the excitons funnel to the large‐*n* domains with bandgap of around 1.64 eV after a short time delay. Normalized bleaching kinetics at 2.18, 1.95, and 1.64 eV following excitation at 400 nm suggest a fast exciton localization time and a relatively slow exciton localization time, indicating that a substantial portion of the photogenerated excitons from the dominant domains of *n* = 2 will be localized to large‐*n* domains within 0.5 ps. Subsequently, a slower decay also matches well with the relative slower formation of the large‐*n* QWs photobleaching. This relatively slower exciton localization time is fitted to be about 50 ps. Similar carrier concentration with enhanced PL intensity has been observed in different 2D RPP polycrystalline films with organic spacers of alkyl ammonium,^[^ ^97^, ^98^ ^]^ phenylethylammonium,^[^ ^99^, ^100^ ^]^ 1‐naphthylmethylammonium,^[^ ^101^, ^102^ ^]^ and phenylbutylammonium.^[^ ^103^ ^]^ Moreover, the directional exciton transfer along the bandgap cascade from small *n* to large *n* phases, converging excitations at the lowest energy phases, resulting in the population inversion for stimulated emission. Thus, optically pumped laser with low thresholds (27–31 µJ cm^−2^) has been fabricated based on (BA)_2_(FA)*_n_*_−1_Pb*_n_*Br_3_ *_n_*_+1_ with average 〈*n*〉 = 3.^[^ ^104^ ^]^

#### 3.2.2 Controversy on Energy Funneling Mechanism: Energy Transfer versus Charge Transfer

Although there is no doubt on the existence of energy funneling in 2D RPP crystals and films, there are yet some challenges need to be further addressed before prompting wider applications. The exact mechanism of energy funneling is still unclear. Charge transfer (CT) and Förster resonance energy transfer (FRET) are the two most possible pathways for the energy funneling. For an energy transfer, the energy cascades from wide‐bandgap domains to the narrow‐bandgap domains via virtual photon at ultrafast timescale (within a timeframe of ps),^[^ ^29^, ^35^, ^99^ ^]^ which relies on the distance, spectral overlap, and dipole orientation between donor and acceptor. Whereas, the CT mechanism suggests the photogenerated charge carriers cascade along with the “ladder‐like” energy levels then ultimately accumulate on the domains with the narrowest bandgap (Figure 4d).^[^ ^29^, ^77^, ^105^ ^]^ For the conventional CT concept, the charge carriers “travel” from a donor to acceptor. Whereas, in the energy funneling system, CT suggests carrier diffusion driven by the concentration gradient and carrier drift driven by a bandgap gradient without the separation of donor and acceptor. At the early stage, the FRET mechanism has been supported by the TA and TRPL spectroscopy, which was widely regarded as the dominant mechanism for the high efficiency of 2D RPPs.^[^ ^29^, ^35^, ^99^ ^]^

However, Chen et al. stressed the FRET rate (*k*_ET_) should be very low.^[^ ^106^ ^]^ The *k*_ET_ is inversely proportional to the dielectric constant. Since the dielectric constant of the inorganic perovskite layer (*ε*_perovskites_ = 25–32)^[^ ^107^ ^]^ is much higher than that of organic spacer (*ε*_organic_ < 3),^[^ ^108^ ^]^ blocking the FRET (Figure 4d). Then, steady‐state and transient PL spectra were separately measured under electrical injection in a hole‐only and electron‐only devices, respectively, verifying the CT mechanism. Furthermore, the photoexcitation intensity dependent PL spectra also revealed that the CT mechanism is more important than ET mechanism. The model of CT is also supported by Liu et al. on energy funnelling in (BA)_2_(MA)*_n_*_−1_Pb*_n_*I_3_ *_n_*_+1_.^[^ ^109^ ^]^ Based on static and transient PL and TA spectral analysis, it is found that photoinduced electron consecutively transfers from small‐*n* to large‐*n* phases while hole transfer on the opposite directions on the timescale of hundreds of picoseconds, which is driven by the band alignment between 2D RPP phases. Consequently, accumulations of electrons and holes on the spatially separated upper and bottom surfaces of the films were observed. This kind of internal charge separation can principally facilitate the charge extraction to the electrodes when applied in the solar cell. This p–n junction‐like carrier dynamics paves the way for their novel applications in optoelectronic devices.

In addition to FRET and CT, photon recycling is another possible pathway for the energy funneling,^[^ ^110^, ^111^ ^]^ but it has not been fully considered yet. We would like to emphasize that the energy funneling may be further engineered for diverse optoelectronic applications when the controversy on mechanism is clearly clarified. If the energy funneling is dominated by FRET, it is much faster than nonradiative trappings thus avoiding the loss to nonradiative centers. Targeting on LEDs, the composition of 2D RPPs can be tailored to orientate the energy transfer into the lowest‐bandgap minority phase, which could be realized by slowing the formation of the pure *n* = 1 phase to improve the monodispersity of *n* = 2, 3, 4 phases. On the contrary, if CT is mainly responsible for the energy funneling effect, the electrons and holes can be separately concentrated, which will bring benefit to the high‐performance photovoltaics (PV) and photodetectors. Very recently, a spectroscopic technique is designed and developed to distinguish the FRET and CT during the energy funneling process by comparing PL spectra excited by pulsed and CW lasers, which has been verified in the FAPbBr_3_/FAPb(Br*_x_*I_1−_ *_x_*)_3_/FAPbI_3_ composite MPs synthesized by a two‐step CVD method.^[^ ^112^ ^]^ This spectroscopic method may be extended to explore the energy funneling mechanism in 2D RPPs.

### 3.3 Giant Rashba Splitting Due to Strong Spin–Orbit Coupling (SOC)

**Figure 5:**
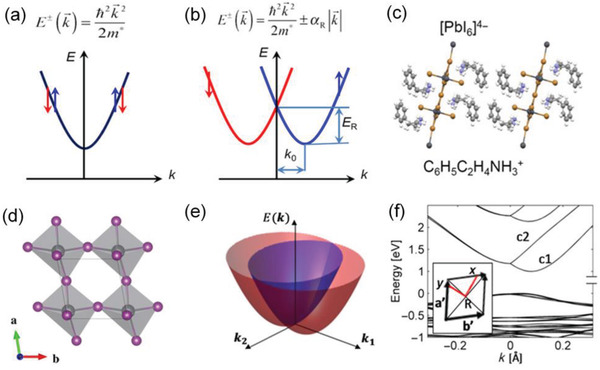
a) Electron dispersion of a regular conduction band (CB) without the Rashba effect. b) Electron dispersion of a CB with the Rashba splitting. The spin‐degenerate bands split into two spin‐polarized bands. c) Structural scheme of (PEA)_2_PbI_4_. d) The Pb atom (gray sphere) is displaced from the octahedra center, breaking off the inversion symmetry, leading to the Rashba splitting. e) Illustration of the CB near the R point in the Brillouin zone. f) The Rashba splitting demonstrated by the calculated electronic structure near the R point. Reproduced with permission.^[^ ^117^ ^]^ Copyright 2017, American Association for the Advancement of Science.

Rashba effect established from strong spin–orbit coupling (SOC) with structure inversion asymmetry is a significant effect that intrinsically affects the carrier dynamics, so as the performances of light emission and photovoltaics. As schemed in **Figure** **5**, the electron dispersion relation, *E*(*k*) is usually described by the effective‐mass approximation, with a spin‐degenerate parabolic dispersion, *E*(*k*) = ℏ^2^ *k* ^2^/2*m* ^·^ , where m* is effective mass of electrons and holes. Whereas, the spin‐degenerate bands can be split into two spin‐polarized bands due to SOC in noncentrosymmetric compounds, known as the Rashba effect.^[^ ^113^, ^114^ ^]^ The electron (or hole) dispersion relation is described by *E_z_*(*k*) = ℏ^2^ *k* ^2^/2*m* ^·^ *z* *α*_R_|*k*|, where *α*_R_ is the Rashba splitting parameter. The two Rashba splitting branches have opposite spins, which can directly influence the optical absorption, carrier relaxation, and magnetic properties, indicating enhanced performance in PV and potential applications in spintronics.

The Rashba effect has been extensively discussed in organic–inorganic hybrid LHPs. For instance, Zheng et al. measured a Rashba effect prolonged carrier lifetime in 3D MAPbI_3_ materials.^[^ ^115^ ^]^ They demonstrated that the carrier recombination rate is decreased due to the spin‐forbidden transition. The 3D Rashba effect including spin degrees of freedom offers a new paradigm for photoelectronic applications. It is believed that in 2D RPPs, the Rashba effect could be enhanced due to the enhanced SOC in reduced dimensions.^[^ ^116^ ^]^ Zhai et al. showed that the 2D perovskites (PEA)_2_PbI_4_ possess giant Rashba splitting.^[^ ^117^ ^]^ From the peak of the exciton transition determined by picosecond transient photomodulation and the free carrier absorption determined by steady‐state photomodulation, they found the 2D (PEA)_2_PbI_4_ exhibits a Rashba parameter of 1.6 eV Å and a large Rashba splitting energy of *E*_R_ = (40 ± 5) meV, which is among the biggest values reported so far. However, on the contrary, Park et al. demonstrated the 2D (PEA)_2_PbI_4_ is a centrosymmetric crystal, since no difference between left and right circular‐polarized emissions was observed at room temperature, indicating Rashba splitting is negligible in (PEA)_2_PbI_4_.^[^ ^118^ ^]^ Moreover, they reported 2D Dion–Jacobson (DJ) phase 4‐(aminomethyl) piperidinium (AMP) lead iodide perovskites (AMP)PbI_4_ exhibit robust ferroelectricity and large Rashba effect with an energy splitting of 85 meV and Rashba coefficient of 2.6 eV Å.^[^ ^118^ ^]^ Therefore, the investigations and conclusions on Rashba effect of 2D perovskites are still under debate.

### 3.4 Strong Nonlinear Optical (NLO) Properties

#### 3.4.1 Third‐Harmonic Generation

Recently, large optical nonlinearities (NLO) have been extensively observed in many inorganic 2D materials, such as graphene oxide,^[^ ^119^ ^]^ MoS_2_/MoSe_2_,^[^ ^120^ ^]^ WS_2_/WSe_2_,^[^ ^121^ ^]^ hexagonal boron nitride (h‐BN),^[^ ^122^ ^]^ and black phosphorus (BP).^[^ ^123^ ^]^ It has also been reported that the nonlinear optical response can be significantly enhanced by thickness tuning.^[^ ^124^, ^125^ ^]^ In the 2D RPP QWs, there are large populations of thermally stable and delocalized excitons, which can strongly interact to make them deviate from ideal harmonic oscillators.^[^ ^126^, ^127^ ^]^ Therefore, the 2D RPPs are expected to exhibit strong third‐order optical nonlinearities under resonant excitation.^[^ ^124^, ^125^ ^]^ Measurements on third‐harmonic generation (THG) of 2D RPPs have been carried out on polycrystalline films and powders.^[^ ^21^, ^46^, ^47^, ^127^ ^]^ However, only weak THG has been reported due to the limited coherent length of the 2D excitons caused by grain boundaries and defect states in these bulk polycrystallines.^[^ ^21^, ^127^ ^]^ To observe the enhanced THG, Abdelwahab et al. performed THG nonlinear optical measurements on 2D RPP nanosheets mechanically exfoliated from large‐size 2D RPP single crystals of (C_4_H_9_NH_3_)_2_PbBr_4_ (*n* = 1), (C_4_H_9_NH_3_)_2_PbI_4_ (*n* = 1), (C_4_H_9_NH_3_)_2_(CH_3_NH_3_)Pb_2_I_7_ (*n* = 2), and (C_4_H_9_NH_3_)_2_(CH_3_NH_3_)_2_Pb_3_I_10_ (*n* = 3).^[^ ^128^ ^]^ h‐BN encapsulation was added to avoid sample degradation during the optical measurements. They observed ultrastrong THG with a maximum effective third‐order susceptibility (*χ* ^(3)^) of 1.12 × 10^−17^ m^2^ V^−2^ with THG conversion efficiencies up to 0.006% for *n* = 2 lead iodide based RPP with thicknesses less than 100 nm. The high conversion efficiency of 0.006% is more than 5 orders of magnitude higher than previously reported values for inorganic 2D materials.^[^ ^119^, ^120^, ^121^, ^122^, ^123^ ^]^

#### 3.4.2 Second Harmonic Generation (SHG)

**Figure 6:**
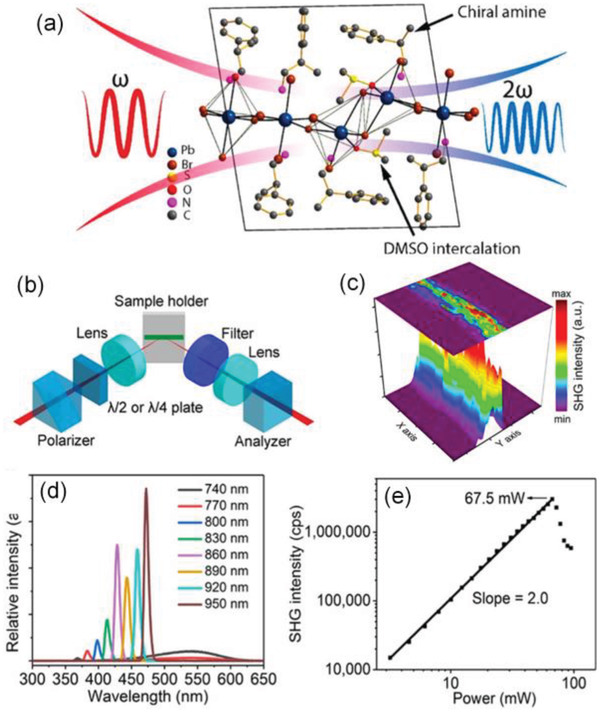
a) Crystallographic structure of the chiral perovskites and illustration of SHG. b) The measurements of SHG. c) The SHG signal scanning of a typical (*R*‐MPEA)_1.5_PbBr_3.5_(DMSO)_0.5_ nanowire. 20 × 20 µm^2^. d) SHG spectra pumped at different wavelengths, which are calibrated by the incident power. e) The relationship between SHG intensity and incident power. Excitation: 850 nm and detection wavelength: 425 nm. Reproduced with permission.^[^ ^132^ ^]^ Copyright 2018, American Chemical Society.

Due to the intrinsic centrosymmetric structures, the studies on the optical nonlinearities of 2D RPPs were mainly focused on the third‐ and higher‐order NLO effects.^[^ ^127^, ^128^, ^129^ ^]^ Strong second‐order NLO responses are hardly expected from general 2D RPPs. However, the modulation of the bulky organic site provides an approach for the functionalization of the 2D perovskites. The structural symmetry can be regulated by changing diverse ammonium groups,^[^ ^130^, ^131^ ^]^ for example, introducing chirality into RPPs by employing chiral amine as the organic component. Inspired by these pioneering works, Yuan et al. developed a new chiral 2D RPPs by using the chiral *β*‐methylphenethylamine (MPEA) as the organic component (**Figure** **6**).^[^ ^132^ ^]^ The noncentrosymmetric assembly of the 2D inorganic layers with a formula of (MPEA)_1.5_PbBr_3.5_(DMSO)_0.5_ in a chiral P1 space group was acquired, where DMSO is dimethyl sulfoxide. And then nanowires of this new 2D RPPs were grown by the antisolvent assisted crystallization in a ternary solvent system. Strong responses of SHG with high polarization ratios were observed from nanowires of this chiral 2D perovskites. Therefore, the introduction of diverse ammonium groups as the organic spacers affords a versatile platform for the functionalization of 2D perovskites, endowing them flexible NLO properties, which are highly desirable for advancing their applications in the next generation integrated photonic circuits.

### 3.5 Spin Control in 2D Chiral RPPs

The control of spin state that is to control the number of electrons in the well‐defined spin state, which could be applied for spintronic devices, such as quantum computing, nonvolatile memory devices, promoting the quantum information technologies. Therefore, the control of spin state is highly desirable. For the semiconductors with large SOC, the electrons occupy the spin‐polarized states under excitation of spin‐polarized (SP) light. Consequently, angular momentum of excitation is converted to spin of electrons, producing electrons spin accumulation, which can be detected by spin‐polarized photoluminescence (SPPL). The strong SOC and giant Rashba splitting make 2D chiral perovskites promising candidates for spintronic devices. Chiral 2D RPPs with 〈*n*〉 = 1 and 〈*n*〉 = 2 were synthesized by incorporating chiral organic ligands *R*‐ and *S*‐methylbenzylammonium bromide (*R*‐MBABr and *S*‐MBABr). Long et al. demonstrated that the chiral 2D RPPs exhibit both circular dichroism and SPPL in the absence of an applied magnetic field, indicating the control of spin state without magnetic field.^[^ ^133^ ^]^ For comparison, a strong external magnetic field of 5 T is required to acquire the same degree of PL polarization in their 3D counterparts. This finding reveals that the chiral 2D perovskites will be promising semiconductors for spintronics.

Moreover, Jana et al. constructed chiral 2D RPPs using chiral, *R*‐ or *S*‐1‐(1‐naphthyl)ethylammonium as spacer cations.^[^ ^134^ ^]^ And they observed a structural chirality transfer across the organic–inorganic interface in these 2D RPPs. The interaction between asymmetric hydrogen‐bonding and lead bromide‐based layers leads to symmetry‐breaking helical distortions in the inorganic layers and Rashba–Dresselhaus spin‐splitting. Chiral 2D RPPs are supposed to exhibit chiroptical activity. And the chiroptical properties can be regulated by modifying the chemical‐composition and structures of the 2D RPPs.^[^ ^135^ ^]^ On one hand, the wavelength of circular dichroism (CD) can be tuned by adjusting the excitonic band structure. For example, the CD signal shifts from 495 to 474 nm when changing the ratio of bromide and iodide anions in *S*‐ or *R*‐C_6_H_5_CH_2_(CH_3_)NH_3_)_2_PbI_4(1−_ *_x_*_)_Br_4_ *_x_*. On the other hand, the CD signal can be switched on/off by changing the crystalline structure.

### 3.6 Transient Energy Reservoir and Exciton Fine Structure

**Figure 7:**
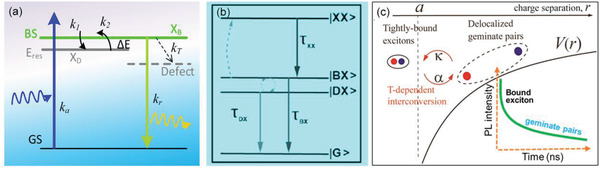
a) Schematic of exciton dynamics with a transient energy reservoir. *E*_res_: energy reservoir, GS: ground state, BS: bright excited sate, *X*_B_: bright exciton, and *X*_D_: dark exciton. Reproduced with permission.^[^ ^136^ ^]^ Copyright 2019, Wiley‐VCH GmbH. b) Proposed energy level diagram for (PEA)_2_PbI_4_ involving a bright exciton (BX) level and a biexciton level (XX), as well as a dark exciton (DX). Reproduced with permission.^[^ ^137^ ^]^ Copyright 2020, Wiley‐VCH GmbH. c) The “reservoir” states are explained by the delocalized geminate pairs, which provide a delayed feeding into the emitting excitons, thus giving rise to the longer‐time PL decay components. Reproduced with permission.^[^ ^138^ ^]^ Copyright 2020, American Chemical Society.

The impact of Rashba splitting on carrier dynamics is also an intriguing topic. Several sets of anomalous photophysical behaviors were observed in (BA)_2_PbI_4_ (BA = CH_3_(CH_2_)_3_NH_3_) crystals,^[^ ^136^ ^]^ which show deviation from the general model of carrier dynamics with irreversible trapping. These observations include 1) two strikingly different PL lifetimes but with high internal quantum efficiency, 2) PL intensity increases with temperature rises from 20 to 50 K, and 3) splitting of the photoinduced bands in transient absorption. As illustrated in **Figure** **7**a, a dark state that acts as special transient energy reservoir was proposed to rationalize the anomalous behaviors. Upon optical excitation, the bright excitons rapidly relax into the low‐lying dark energy reservoir before nonradiative recombination, which can outcompete and avoid nonradiative loss in 2D RPPs. Moreover, the energy trapped by the reservoir is not lost. The energy reservoir can spontaneously recovery the trapped energy to the bright states, thus it can still effectively contribute to the PL and photonic‐electronic conversion. Importantly, all the anomalous observations are well rationalized by the energy reservoir model. However, the nature of dark energy is still elusive. The spin forbidden state caused by the Rashba effect was tentatively proposed as the nature of dark state, implying the carrier dynamics is significantly affected by Rashba splitting. However, the dark exciton is not able to be directly detected, the nature of the reservoir cannot be clearly determined. Other forms of dark excitons could probably be proposed if further evidence is available in the future. The transient energy reservoir based on exciton fine structure provides a novel insight into the mechanism for the lauded defect tolerance of 2D RPPs as a possible pathway for the high efficiency energy transformation for PV and photonics. The exciton fine structure with dark state is also supported by Fang et al.^[^ ^137^ ^]^ A similar bright/dark splitting energy of 10 meV was estimated by transient PL at low temperature in high‐quality single crystals, which was attributed to the electron–hole exchange interaction (Figure 7b).

A reservoir like behavior was also observed by Mondal et al. in quasi‐2D (EN)_4_Pb_2_Br_9_·3Br perovskite (ethylene diammonium, EN).^[^ ^138^ ^]^ The carrier kinetics were studied across a range of excitation fluences and temperatures. It is found that the PL dynamic is substantially *T*‐dependent over the whole range of 77−350 K, while the PL QY keeps nearly *T*‐independent up to 280−290 K with precipitous decrease at higher temperatures only. These observations suggest the coexistence of emissive and nonemissive species that can convert into each other. In the 2D systems, besides the strong excitonic binding, the increased effects of the electron interaction with the surroundings is also expected, such as self‐trapped excitons^[^ ^139^, ^140^ ^]^ and charge carriers (electron and hole polarons).^[^ ^141^, ^142^, ^143^ ^]^ As well accepted, the spatially separated geminate charge pairs (e.g., electron– and hole–polarons as a consequence of the strong interaction with the environment), could play a role of nonemissive excitation species, which can present at lower temperatures. Whereas, the tightly bound electron−hole pair in the excitonic state would represent an emissive species. The dynamic interconversions between the loosely bound geminate charge pairs and tightly bound excitons lead to the excitation fluence and temperature dependent behaviors (Figure 7c). Intriguingly, the geminate charge pairs that can undergo both recombination and spatial separation, which play the role of “reservoir” states providing a delayed feeding into the emitting exciton.

### 3.7 Modulation of Carrier Dynamics by Organic Spacers

#### 3.7.1 Modulation of Hot‐Carrier Cooling Time and Carrier Lifetime by Organic Spacers

**Figure 8:**
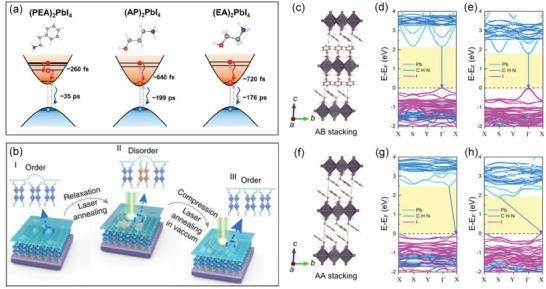
a) Hot electron relaxation and electron−hole pair recombination processes of (PEA)_2_PbI_4_, (AP)_2_PbI_4_, and (EA)_2_PbI_4_ after high‐energy excitation. Reproduced with permission.^[^ ^145^ ^]^ Copyright 2019, American Chemical Society. b) Schematic diagram showing the laser induced order–disorder transition.^[^ ^148^ ^]^ Copyright 2018, Springer Nature. Crystal structures and band structure of (PEA)_2_PbI_4_ with different benzene ring stackings, c–e) for AB stacking and f–h) for AA stacking. (d) and (g) represent the band structures without pressure while (c) and (h) represent the band structures under pressure of 7 GPa. Reproduced with permission.^[^ ^149^ ^]^ Copyright 2019, American Chemical Society.

Hot‐carrier (HC) cooling is a critically important photophysical process that significantly affects the optoelectronic performance. For example, a prolonged HC cooling period could be applied in high‐efficiency hot carrier solar cells, a concept for next‐generation PV devices.^[^ ^144^ ^]^ Yin et al. demonstrated that in the 2D RPP crystals, the HC cooling time strongly relies on the dielectric constant of the organic spacer.^[^ ^145^ ^]^ They found that (EA)_2_PbI_4_ (EA = HOC_2_H_4_NH_3_^+^) with a higher dielectric constant organic spacer has a longer HC cooling time compared to that of (AP)_2_PbI_4_ (AP = HOC_3_H_6_NH_3_^+^) and (PEA)_2_PbI_4_ (**Figure** **8**a). The intraband relaxations of hot carriers in the 2D RPP crystals are strongly influenced by the dielectric confinements due to different polarizations of the inorganic layers and organic spacers. In addition to the dielectric confinements, the slow HC relaxation process is ascribed to a strong screening of the Coulomb interactions, a small nonradiative internal conversion within the conduction bands, as well as a weak electron−phonon coupling, and suppressed rotations of the organic spacers. This work provides a new method for achieving long‐lived hot carriers in 2D RPPs, by using organic spacers with high dielectric constant.

In fact, the choice of the organic spacers in the 2D RPPs also can be adopted to control the charge recombination rate and modulate the performances of photoelectronic devices. It has been shown in several reports that bulky organics tend to rotate in the lattice causing strong electron–phonon interactions and charge localization that improves the emission efficiency of 2D RPPs.^[^ ^66^, ^146^ ^]^ For example, Tsai et al. found that layered 2D RPP LEDs with benzyl ring as organic spacers exhibit a longer radiative recombination lifetime, compared with 2D RPPs containing much shorter alkyl chain spacers.^[^ ^147^ ^]^ As a result, the PL intensity is enhanced by 7.4 times accordingly.

#### 3.7.2 Modulation of Bandgap by Reversible Engineering on Organic Cations

The dynamic modulation of bandgap by reversible engineering on organic cations is also available in 2D RPPs. Leng et al. demonstrated a reversible shift in excitonic energies induced by laser annealing on exfoliated butylammonium based 2D RPP layers encapsulated by hexagonal BN.^[^ ^148^ ^]^ At the initial stage (stage I), the exfoliated 2D RPP layers exhibit a sharp PL peak originating from single excitonic state. Whereas, overexposure to a laser, the sharp PL peak gradually broadens (stage II), and then it redshifted to a final state with less broad feature (stage III). As illustrated in Figure 8b, the redshift and broadened PL bands are explained by surface relaxation effects. The laser annealing induces orientation disorder of the surface organic cations, thus trap states are created. Consequently, after laser annealing, emissions from both band edge excitonic state and radiative surface states are included, leading to the broadened PL peaks. When laser annealing further continues, the PL mainly stems from trap states, thereby the PL peak position redshifted with a less broad width. Intriguingly, due to the protection by the BN layer, the disorder organic cations on perovskites are prevented from desorbing, thus these defects can be thermally cured. When the exfoliated 2D RPP layer is exposed to a high‐power laser under vacuum, the PL evolution can recover from stage III to the initial stage I. Unexpectedly, the laser annealing induced shallow trap states can improve the photogain in a photodetector. Since the trapping states, similar to the self‐doping effect, are selective for specific charges. If the holes are trapped by the surface relaxation states, the electrons are allowed to diffuse/drift a longer distance.

Moreover, the electronic structures and carrier dynamics are also strongly dependent on the stacking order of organic cations, which can be modulated by pressure. For example, in the (PEA)_2_PbI_4_, there is a benzene ring in the PEA cation. As illustrated in Figure 8c, the benzene ring in initial direct bandgap (PEA)_2_PbI_4_ is in AB stacking while the stack order changes to AA stacking and the bandgap becomes indirect under high‐pressure. It is reported that a reversible direct‐indirect band gap transition occurs under pressure at around 5.8 GPa, indicating an additional degree of control for the carrier relaxations of 2D RPPs.^[^ ^149^ ^]^

### 3.8 Anomalous Stark Effect

#### 3.8.1 Anomalous Quantum‐Confined Stark Effect (QCSE)

The QCSE refers the change of optical absorption or emission when an external electric field is applied on a quantum well. Due to oblique of energy level caused by the external electric field, the electrons move to lower energies, while the holes shift to higher energy states, normally leading to a redshift of absorption or emission. The 2D layered RPPs are regarded as analogues of quantum wells, thus, QCSE is potentially expected. Walters et al. fabricated modulators by sandwiching the 2D layered RPPs films between transparent top electrode (indium tin oxide coated glass) and reflective bottom electrode (Ag) to investigate the electroabsorption (EA) spectroscopy.^[^ ^150^ ^]^ QCSE was studied by collecting electrical field‐induced changes of light absorption at room temperature. Accidentally, an anomalous blue‐shifting is observed for methylammonium cations based 2D RPPs. When 2D RPPs are subjected to an electrostatic field, rotation of the methylammonium leads to the separation of the electron and hole wavefunctions, resulting in diminution in the exciton binding energy. When the opposing changes in exciton binding energy are larger than the Stark shifts for *n* = 3 and 4 RPPs, a net blue‐shifting QCSE is observed. The absorption coefficient changes of solution‐processed 2D RPPs is roughly one order of magnitude higher than those for the Franz‐Keldysh‐Aspnes effect of bulk 3D perovskites,^[^ ^151^ ^]^ comparable to those of epitaxial compound semiconductor heterostructures.

Anomalous QCSE was also investigated in the fluorous‐2D perovskites ((Fluo)_2_PbI_4_) by Queloz et al., where Fluo = (CF_3_)_3_CO(CH_2_)_3_NH_3_^+^.^[^ ^152^ ^]^ According to the framework of Stark's theory, the EA was decomposed into a linear combination of first‐ and second‐derivative contributions to the linear absorption spectrum. The first‐derivative proportion of redshift is assigned to QCSE, while the second derivative contribution suggests the formation of screened electron–hole pairs. As evidenced by the anomalous second derivative shape of the Stark signal, they concluded the existence of long‐lived weakly bound charge pairs in layered *n* = 1 (Fluo)_2_PbI_4_ 2D perovskites, which is rationalized by the combination of the electrostatic and structural distortion effects.

#### 3.8.2 Spin‐Selective Optical Stark Effect

**Figure 9:**
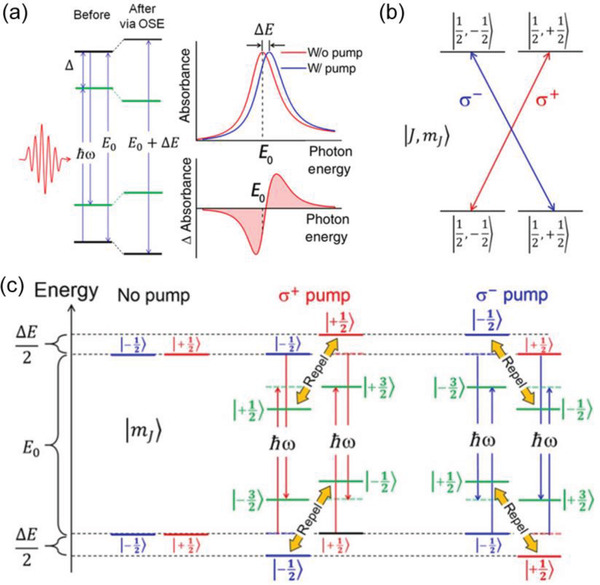
a) Diagram of OSE in a two‐level system. The equilibrium states and the pump‐induced Floquet quasi‐states are represented by black and green lines, respectively. The corresponding linear absorption (upper) and TA spectra (lower) are shown in the right panel. b) Diagram of the optical selection rule. c) Illustration of spin‐selective OSE in 2D RPPs. The same magnetic quantum number (*m_J_*) of equilibrium states and the Floquet quasi‐states leads to the repulsion. The energy levels before and after repulsion are represented by dash and solid lines, respectively. Reproduced with permission.^[^ ^153^ ^]^ Copyright 2016, American Association for the Advancement of Science.

Giovanni et al. reported a strong ultrafast spin‐selective optical Stark effect (OSE) in solution‐processed (C_6_H_4_FC_2_H_4_NH_3_)_2_PbI_4_ RPP thin films.^[^ ^153^ ^]^ As illustrated in **Figure** **9**, OSE refers to a coherent, nonlinear light‐matter interaction arising from the coupling between photons and electronic states. Based on measurements of circularly polarized TA spectroscopy, the spin degeneracy lifting via OSE of (PEA)_2_PbBr_4_, (PEA)_2_PbI_4_, and (C_6_H_4_FC_2_H_4_NH_3_)_2_PbI_4_ are determined to be 1.2 ± 0.3, 4.5 ± 0.3, and 6.3 ± 0.3 meV, respectively, and the corresponding Rabi energy are 33 ± 3, 47 ± 2, and 55 ±2 meV. This large magnitude of energy separation of (C_6_H_4_FC_2_H_4_NH_3_)_2_PbI_4_ is equivalent to Zeeman effect with an applied magnetic field >70 T, which is much larger than the conventional semiconductors. With the additional spin degree of freedom, the OSE exhibits untapped applications for ultrafast optical implementations of quantum information applications.

## 4 Conclusions and Outlook

2D RPPs with unique photophysics have recently gained growing interest as candidates for the next generation optoelectronic devices for their improved stabilities under ambient conditions. However, current studies and review on 2D RPPs are mainly focused on their linear photophysics properties, with relatively rare reports on their quasiparticle dynamics, anisotropy, transient photophysics, nonlinear, and spin‐related properties. Due to the absence of connectivity between the [BX_6_] octahedra, the dimensionality of electronic structures reduces, leading to the localization of carriers. The effective masses of carriers are larger than those of the 3D counterparts. The unique excitonic properties, with large exciton binding energies and high oscillator strengths, enable strong electron–phonon coupling, and polarons of 2D perovskites. In recent years, 2D RPPs with different structures are synthesized and diverse novel photophysics have been successively excavated in 2D perovskites, which are highlighted in this review to inspire future developments of solar cells, LEDs, photodetectors, lasers, spintronics, quantum information, integrated photonic chips, and beyond.

The research on photophysics of 2D RP perovskites is still in their infancy although significant efforts and progresses have been made. There are still many debates that required further clarifications. For example, the energy funneling effect plays a critical role on the high‐efficient light emission. However, as discussed in Section 3.2.2, both energy transfer and charge transfer are controversially proposed to be responsible for energy funneling mechanism. A clear understanding on this issue will form guidelines to control the sizes and distributions of the domains with different *n* values to pursuit a higher emission efficiency. For another example, as reviewed in Section 3.4, the conclusions on Rashba splitting of (PEA)_2_PbI_4_ perovskites are even converse. Therefore, the existence of Rashba effect in specific 2D RPPs is still a controversy. Moreover, the impact of Rashba effect on the carrier dynamics and the detailed origins of the observed Rashba effect are still unclear. There is still a lack of effective approaches to modulate the amplitude of Rashba splitting. Therefore, more investigations on the photophysics are still highly desirable to clarify these controversies.

Unlike the conventional inorganic QWs, the organic cationic ligands in the 2D RPPs can be flexibly modified. The excitons are confined within the inorganic layers, due to the energetic barriers of low dielectric screening from the surrounding organic spacers. Such quantum confinement effect including band structure, exciton binding energy, and exciton recombination rate can be regulated by changing the organic cationic ligands with different lengths, dielectric constants, and stacking order. Moreover, structural symmetry of the 2D RPPs can be controlled by introducing chiral organic cationic spacers or aligning the dipolar organic cationic spacers, so that Rashba effect, nonlinear optical properties, spin‐selective optical Stark effect can be introduced. The design of organic sites has afforded a versatile platform for the modulation and functionalization of the 2D RPPs. Therefore, more future research efforts could be devoted to engineering the organic cationic ligands.

In terms of functional devices, controllable carrier/exciton/energy transport may be designed by heterojunctions of 2D RPPs with different *n*, or with other 2D atomic layers, which will boost the optoelectronic performances. Moreover, the chiral 2D RPPs have shown prospect as spintronic semiconductors. The function of chirality by organic cationic spacers, combining with the excellent optoelectronic properties, will increasingly attract interest in the field of spintronics. Spin‐polarized excitons generated in chiral 2D RPPs might be utilized to construct the optically controlled “spin‐torque” switches, which will revolutionize future quantum information technologies, such as quantum computing, nonvolatile memory devices, and photonic chips etc. The spin‐related optical behaviors of chiral 2D perovskites deserve more attention in the future. It is of great significance to promote their untapped potential beyond LEDs and photovoltaics.

## Conflict of Interest

The authors declare no conflict of interest.
